# Aging with long-term physical disability: Cohort analysis of survey sample in the U.S.

**DOI:** 10.12688/f1000research.74532.1

**Published:** 2022-01-20

**Authors:** Kerri A. Morgan, Michelle Putnam, Sandra M. Espin-Tello, Marian Keglovits, Margaret Campbell, Yan Yan, Aimee Wehmeier, Susan Stark

**Affiliations:** 1 Program in Occupational Therapy, Washington University School of Medicine, St. Louis, Missouri, 63110, USA; 2 School of Social Work, Simmons University, Boston, Massachusetts, 02115, USA; 3 EGOKITUZ, Universidad del País Vasco/Euskal Herriko Unibertsitatea, Lejona, Bizkaia, Spain; 4 Campbell and Associates Consulting, Seattle, Washington, USA; 5 Department of Surgery, Washington University School of Medicine, St. Louis, Missouri, 63110, USA; 6 Paraquad, St. Louis, Missouri, 63110, USA

**Keywords:** aging with disability, physical disability, cohort, health, participation

## Abstract

**Background:** Chronic health conditions, secondary conditions, and decreasing functional ability related to aging and/or changes in underlying impairment may influence participation for persons aging with long-term physical disability (AwD).

**Objective:** To examine sample integrity and baseline findings through exploration of associations of sociodemographic, health, and disability factors with social participation for persons AwD.

**Methods:** This is a longitudinal cohort study following persons AwD over three years, reporting baseline cohort study data. A convenience sample of 474 persons AwD aged 45–65 reporting physical disability of ≥5 years’ duration was recruited through community organizations and social media. The cohort was majority female (66.7%) and single (62.0%), and over one-third (38.6%) was non-White. Pain, fatigue, depression, ability to participate in, and satisfaction with, social roles and activities were measured with the Patient Reported Outcomes Measurement Information System.

**Results:** Participants aged 55–60 and 61–65 had significantly lower rates of employment and marriage and higher rates of living alone than participants aged 45–54. Participants reported higher rates of fatigue, pain, and depression and lower ability to participate in, and satisfaction with, participation in, social roles and activities than the general population. Ability to participate and satisfaction with participation were highest among Black/African American participants.

**Conclusions:** Participants reported higher rates of common AwD symptoms and lower ability to participate and satisfaction with participation than the general population, consistent with prior studies of AwD samples. This cohort reflects the AwD population and can be considered an AwD sample, comparable to those found in existing literature. The focus of future analyses will be to gain a greater understanding of chronic health conditions, incidence of falls, engagement in everyday life activities, and the impact of the environment.

## Introduction

Aging with a disability is the phenomenon of living long-term with impairment and disability that begins in early and/or mid-life and continues over the lifecourse. ^1^^,^^2^ Although investigation of aging with long-term physical disability (AwD) dates back over 30 years, to date, most of the research has been small-scale and focused primarily on individuals with lifelong and early-onset disabilities. ^3^^–^^5^ In contrast, our research focuses on persons AwD between the ages of 45 and 65 living in the United States, with disability onset from birth to age 60, to understand participation patterns and changes in this mid-life phase. It is fairly well-established that these individuals often experience the aging process earlier, at a faster rate, and report greater difficulty with independent living than their peers without physical disabilities. ^6^^,^^7^ The growing body of research related to AwD links disability-related fatigue, pain, and depression to problems participating in general or to reduced social participation. ^8^^–^^10^ Existing studies have also shown that increased levels of functional impairment, secondary health conditions (e.g., depression, fatigue, and sleep disturbance), and reduced mood and energy related to secondary health conditions significantly decrease satisfaction with social roles among persons AwD. ^11^ Additionally, there is a substantial body of evidence showing differences in the severity of disability and disability symptoms and trajectories among individuals in mid-life based on race and ethnicity. ^12^^,^^13^ However, limited research has investigated participation among persons AwD as a population group. ^9^ Only a few research studies have explored AwD longitudinally. ^1^^,^^14^^,^^15^ Of those, the Aging and Quality of Life Survey at the University of Washington was the longest running, collecting seven waves of data from 2009–2018. ^16^ That study produced important findings regarding persons AwD and health and wellness but offers limited information about participation. We seek to build on these findings by expanding our understanding into the realm of participation in life activities.

Our three-year cohort survey of persons AwD considers how and why participation changes over time. We aim to inform evidence-based interventions implemented by community organizations and service providers designed to facilitate participation of people with disabilities. Here, we report cohort sociodemographic, health, and disability traits from our first wave of data (collected August 2018 - July 2019) by age group. We also explore sample integrity by considering how our population compares to those existing in the current literature, based on the presence of normative AwD symptoms reported in the literature. The population of persons AwD is not well-represented in national datasets, as very few general population–based surveys in the United States include age of onset of conditions, symptoms, or disability, nor do they typically contain measures of pain, fatigue, and depression, which are often-measured symptoms related to AwD. ^1^^,^^2^ We take this first step to help validate our sample given the paucity of research focused on AwD. We want to ensure that we indeed have recruited a sample of individuals that reflects the traits of AwD samples in published research. There is no standard set of parameters for what makes a sample an AwD sample, versus a more generic sample of persons with disabilities. ^1^^,^^2^ We believe that the presence of AwD symptoms—given the commonality of their existence in the small body of AwD research—and one’s age at disability onset are the best current markers for identifying this subset of the broader disability population.

After we assess demographic and disability characteristics, we then assess ability to participate in, and satisfaction with, participation in social roles and activities to further review our cohort. This will be compared with findings in existing literature, in order to gain a better understanding of baseline social participation prior to the COVID-19 pandemic. Specifically, we identify sociodemographic, health, and disability variables associated with one’s ability to participate in, and satisfaction with, their participation in social roles and activities, and we then quantify the independent effects of the variables on these two aspects of social participation. Our social activity measures are drawn from the PROMIS (Patient Reported Outcomes Measurement Information System) measurement bank and have been evaluated across multiple populations. ^17^^–^^19^

AwD research study samples are frequently composed of persons who identify their race as White and who have a specific diagnosis such as spinal cord injury, multiple sclerosis, or polio, ^20^ which represent some of the common conditions found among persons AwD, but often do not include other conditions that cause disability. Guidelines from the United States federal funding agencies including the National Institutes of Health and the National Institute on Disability, Independent Living, and Rehabilitation Research ^21^ have required researchers to improve the racial and ethnic diversity of their study samples, given the substantial differences in health and wellness outcomes for non-White individuals in the United States. ^22^

In our study, we actively sought to create a broader sample base by increasing the proportion of non-White participants and by recruiting participants based on self-report of physical disability, regardless of diagnosis. We worked closely with our Community-Based Research Network (CBRN), ^23^ a network of aging and disability providers and advocacy groups located in the state of Missouri in the United States, that work together to close the gap in the availability of evidence-based practices for persons AwD. The CBRN supported this cohort by identifying areas of importance for data collection, assisting with recruitment, and strategizing about increasing recruitment of participants of specific groups, including men and non-White individuals. Because of this, our study sample is distinct from other study samples of persons AwD in the literature. This reinforced our interest in evaluating the traits of our sample against those of other samples, before we begin our longitudinal analysis.

Our cohort study is guided by disability models considering person–environment interactions ^24^^,^^25^ and the World Health Organization’s International Classification of Function, Disability, and Health ^26^; Active Ageing policy framework ^27^; and Conceptual Framework for Action on the Social Determinants of Health. ^28^ Fundamentally, our study process and choices for measures are anchored in building our understanding of how the knowledge gained from this study can be useful to CBRN members and other community-based organizations, to support the participation of persons AwD. Finally, our study is situated within the COVID-19 pandemic. The pandemic has exacerbated socioeconomic, health, and independent living disparities for persons with disabilities in the United States. ^29^^,^^30^ Although this cohort study was not intended to evaluate participation among persons AwD before and during a pandemic, it has. Therefore, we believe it is crucial to understand how findings for baseline sample and social participation measures fit within the extant literature, so that we may have a better ability to tease apart the effects of the pandemic from more expected changes over time.

The questions we ask of our sample in this initial analysis are as follows. How do the sociodemographic and disability characteristics of our sample compare with those of other samples in AwD research and with the general population? How do social participation and predictors of social participation in our sample compare with findings in related studies of persons AwD? These questions support our dual aim of understanding how our sample compares with AwD samples reported in the published literature and providing a context for our future longitudinal analysis.

## Methods

### Ethics

Ethics approval was granted by the Washington University Institutional Review Board (IRB) (IRB# 201710186). This study presented minimal risk to participants; therefore, the IRB approved a request to waive documentation of informed consent. Participants were provided the consent information either online or over the phone and were asked if they would like to continue with participation in the study. Only participants who indicated yes online or over the phone continued with the survey and were included in the analysis. The consent information sheet explained that, when writing reports about the study, the research team will do so in a way that participants cannot be individually identified and that information the research team shares will be de-identified.

### Study design

This is a longitudinal cohort study, collecting survey data once a year for three years at 12-month intervals: study enrollment (T0), 1-year follow-up (T1), and 2-year follow-up (T2). Here, we report findings from T0.

### Participants and procedures

To enroll, participants had to be aged 45–65 years, have experienced physical disability for a duration of ≥5 years, speak English, and autonomously provide consent. Purposive recruitment occurred through a CBRN, ^23^ local events, and social media. The organizations and agencies in the CBRN shared IRB-approved information about the study to their clients and participants through e-mail, mailed letters, and/or posts on their social media platforms. Local disability and aging events in the St. Louis, Missouri, area were attended by members of the research team. These events included bus pass distribution events and disability fairs. In addition, a Facebook advertisement was purchased, and an IRB-approved social media statement on the research study was posted for a limited amount of time. Statistical power calculations estimated a T0 sample of 470–500 participants, assuming a 25% attrition rate at T1 and T2. Gift cards were provided for completed surveys.

Recruitment for T0 occurred between August 2018 and July 2019. The eligibility screen was completed by 1254 individuals; 977 passed the screening, and 516 were eligible and agreed to consent. We excluded 42 responses primarily due to duplicate survey completions, meaning participants completed the survey twice, and inconsistencies between screener and survey responses. A total of 474 unique participant responses were valid for analysis at T0. Interested participants used the information distributed for recruitment (a phone number and an e-mail address) to contact the research team to express interest and determine eligibility. To broaden the opportunity for completion, a mixed methods approach of either telephone or online administration of the screener and survey was offered. Half of the participants chose to be screened, provide consent, and complete the survey online, the other half over the phone. All surveys were the same for online and telephone administration and were completed using REDCap (Research Electronic Data Capture; Legacy Version 7 Server), a secure, web-based application. ^31^ A secure link was sent with a password login to participants who selected online administration. These individuals directly recorded their responses into the online REDCap survey. For those who selected telephone administration, an appointment with a trained member of the research team was scheduled. During this appointment, the research team member directly read the questions and response options to the individual and recorded the individual’s responses into the REDCap survey. The average time for completion was 45–60 minutes.

### Measures

The assessments for all three time points consisted of self-reports of health, disability, and social support characteristics; activity, participation, and environmental factors; and long-term service and support use (for the measures used in the survey, see *Extended Data*). Measures were selected in consultation with the CBRN. In this paper, we report the sociodemographic, health and disability, and social participation measures at T0.

Sociodemographic characteristics included age, race/ethnicity, sex at birth, gender, marital status, education, living arrangement, employment status, food security, sources of income, and health insurance. Race and ethnicity were asked as a combined question with response options of White, Black/African American, Hispanic or Latino, Asian/Asian Indian, Middle Eastern, American Indian/Native American, Native Hawaiian or Pacific Islander, and other. Sex at birth had response categories of female, male, intersex, I do not identify with any of these, and prefer not to say. Gender identity responses included man, woman, transgender, none of these describe me, and prefer not to say. Annual income was measured using the individual income eligibility limit at T0 for Missouri’s Medicaid program of $10,008 annually ^32^ for older adults and persons with disabilities not enrolled in the Home and Community Based Services Waiver. ^33^ The response for primary health condition causing physical disability was open-ended; we coded answers categorically based on the Social Security Administration’s (SSA) Listing of Impairments (Part A) for Adults. ^34^ The duration of this condition was reported in years, ranging from birth to age 60. Self-rated physical and mental health were measured on a five-point scale (5 = excellent, 1 = poor).

We employed several measures from the PROMIS ^35^ that have been validated with persons with physical disabilities. ^36^ The PROMIS Physical Function with Mobility Aid Short Form ^35^^,^^37^ measures one’s self-reported capability of standing and moving with and without support. The short form includes a screening item that asks about one’s ability to walk 25 feet with or without support. Based on the participant’s response, some items are skipped. Raw scores were submitted to the HealthMeasures Scoring Service, which calculated t-scores. The score range is 12–58, with higher scores representing better physical function. Three commonly reported AwD symptoms were measured with PROMIS instruments using the computerized adaptive testing (CAT) versions (REDCap Legacy Version 7 Server). The PROMIS Fatigue Profile evaluates a range of fatigue symptoms, from mild feelings of tiredness to an overwhelming sense of exhaustion. ^35^^,^^36^^,^^38^ PROMIS Pain Interference measures the consequences of pain on relevant aspects of a person’s life, including the extent to which pain hinders engagement with social, cognitive, emotional, physical, and recreational activities. ^36^^,^^39^ PROMIS Depression assesses negative mood, views of self, and social cognition, as well as decreased positive affect and engagement. ^36^ These three measures use a five-point scale, with higher scores representing higher levels of the symptom over seven days. T-scores generated from the PROMIS scales are compared against a mean general population score of 50.

We measured social participation using: (1) the PROMIS Adult Ability to Participate in Social Roles and Activities ^40^ CAT version, which is not time-bound and assesses the perceived ability to perform one’s usual social roles and activities, and (2) the PROMIS Satisfaction with Participation in Social Roles and Activities ^19^ CAT version, which assesses self-reported contentment with social roles, such as work and family responsibilities, over the past seven days. Items are reverse-coded so that higher scores represent fewer limitations (i.e., better abilities).

### Statistical analysis

We used SAS/STAT software (version 9.4, SAS Inc., Cary, NC, USA) ^41^ for analysis, setting significance at p ≤ .05. To explore differences by age, we divided participants into three categories (45–54, 55–60, and 61–65) for univariate analysis. We performed bivariate analyses including chi-square tests and analysis of variances (ANOVA) to examine the differences of categorical and continuous variables across age groups. We then explored differences in the two social participation outcome scores by demographics. We examined univariate associations of each participant’s characteristics with the two social participation outcomes. For categorical variables, we examined the means of each outcome in each level of categorical variable, and we used two-sample t tests (two levels of categorical variables) and ANOVA (more than two levels of categorical variables) to test for statistically significant differences.

## Results

The sample was two-thirds female, one participant identified as transgender, and four participants preferred not to answer the question. Participants had a mean age of 56.8 years (SD = 5.6) and a mean of 19.0 years living with their disability (SD = 13.7, range = 5–65). Seventy percent of participants resided in the state of Missouri; the state with the second most participants was Illinois, with 6.5%. Twenty-eight additional states across the United States were represented, each with ≤2% of participants The most frequently self-reported primary causes of physical disability were neurological (37%; e.g., cerebral palsy, multiple sclerosis, spinal cord disorders, traumatic brain injury) and musculoskeletal (26%; e.g., degenerative and osteoarthritis, spinal stenosis, amputation). Conditions related to respiratory (e.g., asthma, COPD, lung disease), endocrine (e.g., diabetes and thyroiditis), and immunological (e.g., rheumatoid arthritis, connective tissue disorders) systems each ranged from 5%–6% representation. Categories ranging between 0.5%–3% representation included the cardiovascular system, special senses and speech, digestive system, and hematological disorders. Eighty percent of participants reported reasons for their primary disability that can be categorized as having one cause of primary disability, based on SSA listing.

Chi-square tests showed that older participants (aged 55–60 and 61–65) had significantly lower rates of employment and marriage, higher rates of living alone, and had lived with their disability for longer ( Table 1). Rates of Medicare, ^42^ Social Security Disability Insurance (SSDI), ^43^ and Social Security retirement ^44^ benefits receipt were also higher among older participants (aged 55–60 and 61–65).

**Table 1.** Sociodemographic profile of sample cohort by age group.

Sociodemographic traits	Total n = 474	Ages 45–54 n = 149	Ages 55–60 n = 178	Ages 61–65 n = 147	*X* ^2^ Test statistic (between age groups) ^†^
	N (%)	N (%)	N (%)	N (%)	
Sex at birth					.956
Male	160 (33.8)	50 (33.6)	59 (33.2)	51 (34.7)	
Female	314 (66.2)	99 (66.4)	119 (66.9)	96 (65.3)	
Gender (missing = 5)					
Man	156 (33.3)	47 (32.4)	60 (33.9)	49 (33.3)	.080
Woman	313 (66.7)	98 (67.6)	117 (66.1)	98 (66.7)	
Race/ethnicity					
White	291 (61.4)	92 (61.7)	111 (62.4)	88 (59.9)	2.58
Black/African American	125 (26.4)	35 (23.5)	46 (25.8)	44 (29.9)	
Other	58 (12.2)	22 (14.8)	21 (11.8)	15 (20.2)	
Marital status					
Currently married/partnered	180 (37.8)	69 (46.3)	58 (32.6)	53 (36.1)	6.82 *
Single/widowed/other	294 (62.0)	80 (53.7)	120 (67.4)	94 (64.0)	
Education (missing = 1)					
High-school diploma or less	138 (29.2)	37 (24.8)	55 (31.1)	46 (31.3)	3.25
Associate degree or some college/advanced training	181 (38.3)	60 (40.3)	62 (35.0)	59 (40.1)	
Bachelor degree/graduate degree	154 (32.6)	52 (34.9)	60 (33.9)	42 (28.6)	
Employment status (missing = 2)					
Paid work, full- or part-time	87 (18.4)	47 (31.5)	26 (14.8)	14 (9.5)	33.75 *
Seeking paid work ^a^	14 (3.0)				
Retired, not seeking work, other	85 (18.0)	19 (12.8)	27 (15.3)	39 (26.5)	
Disability leave	286 (60.6)	78 (52.4)	117 (66.5)	91 (61.9)	
Living arrangement (missing = 1)					
Live alone	197 (41.7)	42 (28.2)	86 (48.3)	69 (47.3)	16.26 *
Live with others	276 (58.4)	107 (71.8)	92 (51.7)	77 (52.7)	
Personal annual income					
$10,008 or less	166 (35.0)	56 (37.6)	59 (33.2)	51 (34.7)	.712
$10,009 or more	308 (65.0)	93 (62.4)	119 (66.9)	96 (65.3)	
Food security					
0 days hungry last month	367 (77.4)	113 (75.8)	137 (77.0)	117 (79.6)	.631
1+ days hungry last month	107 (22.6)	36 (24.2)	41 (23.0)	30 (20.4)	
Sources of income					
Paid employment	107 (22.6)	52 (34.9)	34 (19.1)	21 (14.3)	19.96 *
Unemployment benefits ^a^	6 (1.3)				
Work-related disability benefits	50 (10.6)	13 (8.7)	22 (12.5)	15 (10.2)	1.16
SSDI & Social Security Retirement	341 (71.9)	91 (61.1)	131 (73.6)	119 (81.0)	14.87 *
SSI (federal income supplement)	64 (13.5)	16 (10.7)	27 (15.2)	21 (14.3)	1.48
Veterans’ disability/retirement benefits ^a^	14 (3.0)				
Retirement pension, savings	58 (12.2)	10 (6.7)	23 (12.9)	25 (17.0)	7.43 *
Assistance from family/friends	39 (8.2)	15 (10.1)	14 (7.9)	10 (6.80)	1.09
Health insurance held					
Medicare	275 (58.0)	73 (49.0)	116 (65.2)	86 (58.5)	8.73 *
Medicaid—Missouri Medicaid program	180 (38.0)	55 (36.9)	75 (42.1)	50 (34.0)	2.36
Military healthcare/TRICARE	28 (5.9)	4 (2.7)	9 (5.1)	15 (10.2)	7.90 *
Private health insurance	159 (33.5)	54 (36.2)	54 (30.3)	51 (34.7)	1.39
None ^a^	15 (3.2)				
Years living with disability (mean)	19.0	16.6	19.6	20.7	3.61 *

Note. Total number may be lower for some variables due to missing information. For source of income and health insurance coverage, each response category is considered a single variable.

^*^ p ≤ .05

^†^ Chi-square tests for similarity of frequency distribution of each variable in column one.

^a^ Cell size ≤ 8 participants.

Over half of participants had at least some difficulty with seeing (53%), self-care (52%), or remembering or concentrating (63%); about one-quarter reported difficulty with hearing (25%) or communicating (22%). Ninety-four percent of participants were unable to walk or climb steps or had difficulty doing so. In the past 12 months, approximately 46% of participants reported that their health status had declined, and 54% of participants reported that their ability to do what they wanted to do in their daily lives had decreased. Table 2 presents health and function information for the total sample and differences by age group. Participants’ mean scores of AwD-related symptoms were all above general population averages of 50, with a fatigue mean of 59.0 (SD = 9.3, range = 24.3–84.7), a pain interference mean of 60.4 (SD = 10.0, range = 38.7–83.8), and a depression mean of 54.9 (SD = 10.1, range = 34.2–84.4). We did not find significant differences by age group for health and function measures.

**Table 2.** Health and function by age group.

Health	Total n = 474	Ages 45–54 n = 149	Ages 55–60 n = 178	Ages 61–65 n = 147	*X* ^2^ Test statistic
	N (%)	N (%)	N (%)	N (%)	
Self-rated physical health					
Excellent/very good	45 (9.5)	22 (14.9)	13 (7.3)	10 (6.9)	11.35
Good	122 (25.9)	36 (24.3)	47 (26.4)	39 (26.7)	
Fair	192 (40.7)	62 (41.9)	66 (37.1)	64 (43.8)	
Poor	113 (23.9)	28 (18.9)	52 (29.2)	33 (22.6)	
Self-rated mental health					
Excellent/very good	134 (28.3)	44 (29.7)	43 (24.2)	47 (32.0)	10.13
Good	150 (31.7)	56 (37.8)	52 (29.2)	42 (28.6)	
Fair	154 (32.6)	36 (24.3)	69 (38.8)	49 (33.3)	
Poor	35 (7.4)	12 (8.1)	14 (7.9)	9 (6.1)	
**Function & health**	**Mean (SD)**	**Mean (SD)**	**Mean (SD)**	**Mean (SD)**	**F-test**
Physical function*	35.7 (8.2)	35.3 (8.7)	35.3 (8.1)	36.5 (7.7)	1.07
Fatigue ^†^	59.0 (9.3)	58.4 (10.4)	59.9 (8.9)	58.4 (8.6)	1.44
Pain ^†^	60.4 (10.0)	59.3 (10.5)	61.0 (9.7)	60.8 (9.8)	1.24
Depression ^†^	54.9 (10.1)	54.1 (10.4)	56.0 (10.1)	54.2 (9.9)	1.90

Note. The PROMIS measure scores are computed to a t-score metric where a higher score means better function* or worse health ^†^.

Participants reported lower average ability to participate in social roles (M = 44.1, SD = 9.0, range = 21.5–67.5) and satisfaction with their participation in social roles (M = 43.5, SD = 9.8, range = 22.0–68.7) than the general population (t-score = 50). Table 3 presents data on each social participation measure by sociodemographic characteristics. Both ability to participate in, and satisfaction with, participation in social roles and activities were higher among Black/African American participants in comparison to White participants and those of other races.

**Table 3.** Descriptive statistics and univariate association of categorical variables with PROMIS Participation measures.

	Ability to participate in social roles and activities (Mean ± SD)	t statistics/F statistics	Satisfaction with participation in social roles and activities (Mean ± SD)	t statistics/F statistics
**Categorical independent variables**				
Gender		*t* = 1.29		*t* = 1.57
Man	44.86 ± 8.98		44.41 ± 9.16	
Woman	43.73 ± 8.94		42.91 ± 10.04	
Marital status		*t* = -1.85		*t* = -1.75
Currently married/partnered	43.17 ± 7.88		42.50 ± 8.75	
Single/widowed/other	44.67 ± 9.56		44.05 ± 10.29	
Living arrangement		*t* = 1.76		*t* = 2.41 *
Live alone	44.98 ± 9.69		44.72 ± 10.17	
Live with others	43.47 ± 8.40		42.54 ± 9.36	
Personal annual income		*t* = 2.70 **		*t* = 1.16
$10,008 or less	45.71 ± 10.09		44.23 ± 11.52	
$10,009 or more	43.24 ± 8.20		43.05 ± 8.64	
Food security		*t* = 1.79		*t* = 2.66 **
0 days hungry last month	44.50 ± 8.81		44.10 ± 9.67	
1+ days hungry last month	42.75 ± 9.42		41.27 ± 9.74	
Employment Status		*F =* 2.08		*F = *4.87 **
Paid work, full- or part-time	46.03 ± 7.47		46.40 ± 7.94	
Seeking paid work	43.28 ± 7.07		42.91 ± 9.12	
Retired, not working, other	44.51 ± 9.44		44.71 ± 10.04	
Disability leave	43.50 ± 9.27		42.21 ± 10.03	
Medicare		*t* = 2.33 *		*t* = 0.65
Yes	43.29 ± 9.23		43.22 ± 9.62	
Medicaid (state Medicaid program)		*t* = -1.41		*t* = -0.99
Yes	44.88 ± 9.91		44.07 ± 11.25	
Military healthcare/TRICARE		*t* = -0.02		*t* = -0.13
Yes	43.14 ± 8.96		43.70 ± 8.54	
Private health insurance		*t* = 0.21		*t* = -0.46
Yes	43.98 ± 8.47		43.75 ± 8.99	
No insurance		*t* = 1.75		*t* = 2.71 *
Yes	40.11 ± 6.63		39.92 ± 4.92	
Age (group years)		*F* = 1.81		*F* = 5.44 **
45–54 years old	44.66 ± 9.49		43.34 ± 10.01	
55–60 years old	43.24 ± 8.68		41.94 ± 9.49	
61–65 years old	44.71 ± 8.76		45.36 ± 9.61	
Race		*F* = 12.50 ***		*F* = 5.75 **
White	42.84 ± 8.19		42.75 ± 9.04	
Black/African American	47.39 ± 10.18		45.84 ± 10.97	
Other	43.49 ± 8.27		41.74 ± 9.85	
Education		*F* = 8.14 ***		*F* = 6.60 **
High-school diploma or less	46.20 ± 10.37		44.96 ± 12.17	
Some college/advanced training	42.16 ± 8.47		41.46 ± 8.38	
Bachelor degree/graduate degree	44.62 ± 7.66		44.44 ± 8.44	

Note.

* p < .05;

** p < .01;

*** p < .001.

## Discussion

Findings from our analyses show that our cohort is distinct yet similar to other AwD study populations. Its racial representation of nearly 39% non-White participants is higher than the only other cohort study of persons AwD we are aware of, which was roughly 90% White participants. ^36^^,^^45^ This may be a factor of study location or recruitment strategies. Seventy percent of our participants live in the state of Missouri where our study is based, which has a Black and African American population of approximately 12% overall; this increases to 25%–50% in urban areas. ^46^ Although there are notable exceptions in which studies have actively focused on non-White participants—for example, work by Harrison *et al*. ^47^ focuses on predominantly Latina participants—broader racial and ethnic diversity is a major limitation in existing AwD research. We believe that the diversity of our sample will help broaden the applicability of cohort findings.

Our cohort has similar percentages of college/advanced education (71%) to the US general population (68%). A higher percentage, 62%, are single, compared to roughly 48% of their age-matched general population peers ^48^; a higher percentage also lives alone (41%) compared to their age-matched general population peers (12%–13%). ^33^ Compared to other samples of persons AwD, our cohort has the same proportion of female participants (66.7%); however, fewer individuals are married (37.8%), a higher percentage is financially poor (35%), and a greater percentage (94%) has difficulty walking or climbing steps. ^8^^–^^10^^,^^45^ In general, our cohort members seem to have fewer social, financial, and physical resources than those in other studies. ^13^^–^^15^^,^^29^

The majority of participants in our cohort (71.9%) receive SSDI. For health insurance, 58% have Medicare, ^42^ and 38% have Medicaid. ^49^ We did not find comparable data for SSDI, Medicare, and Medicaid rates in studies with cross-diagnosis AwD samples to compare our results against. Given the low employment rate of our sample, we reviewed SSDI receipt and determined that 88% are insured by Medicare and 43% by Medicaid. At the time of baseline data collection, the state of Missouri, where most participants reside, had not passed the Affordable Care Act’s Medicaid expansion programs, ^50^ suggesting that some participants may have forgone employment in order to retain public health insurance through traditional Medicaid state guidelines. ^51^

Our participants experience common AwD symptoms found in other studies. For example, using a PROMIS Pain measure in a sample of persons with neurological conditions, Molton *et al*. ^45^ found scores of 51.9 for persons aged 45–54 and 51.6 for persons aged 55–64. Using a PROMIS Fatigue measure with the same dataset, Cook *et al*. ^36^ found mean scores ranging from 52.4–58.7, similar to our cohort’s mean score. Amtmann *et al*. ^39^ used a PROMIS measure, and Jensen *et al*. ^52^ used the PHQ-9 (Patient Health Questionnaire-9), ^53^ and both found elevated depression levels compared to the US general population; we found this too. Based on these comparisons, we have confidence that our cohort does reflect the AwD population in regard to the presence of common AwD symptoms. We did not find comparable data for self-rated physical and mental health. Although there were some significant differences between age groups in demographics, including employment, this was not the case for health and function, where age group membership was not significant.

Our analysis of social roles and social activities had similar results to those found in the Aging and Quality of Life Survey and other studies of diagnostic-specific populations ^9^^,^^11^^,^^54^ examining ability to participate and satisfaction with participation. We believe the similarity in findings related to AwD symptoms demonstrates that our cohort is representative of the AwD population. Quite notable, though, is our finding that social participation scores are higher for Black/African American participants compared to White cohort members. We will continue to explore this difference in future analyses.

### Study limitations

Seventy percent of our cohort is from one state in the United States, and the sample is predominantly female; thus, the cohort likely is not fully representative of the AwD population. Racial and ethnic diversity in our sample is primarily limited to Black/African American participants; other groups are underrepresented.

## Conclusions

We believe that our cohort reflects the AwD population and can be considered an AwD sample comparable to those found in existing literature. The findings from this analysis add to the growing body of research that can be used to both better understand AwD in midlife and inform the design of intervention studies and programs aimed at facilitating participation. Our future analyses will further explore social participation, as well as interactions among disability status and chronic health conditions, incidence of falls, influence of environmental factors on participation, engagement in life activities, and associations between use of long-term services and participation for persons AwD. We believe this cohort study will help inform interventions and programs that support persons AwD to engage in their communities as we assess changes in participation over time.

## Data availability

### Underlying data

The underlying data generated and analyzed during the current study cannot be sufficiently de-identified and, therefore, cannot be made publicly available due to ethical considerations. De-identified data could be made available upon reasonable request, for the purpose of further research, via the corresponding author.

### Extended data

The publicly available measures used in the study survey can be accessed via the links below:

• PROMIS Physical Function with Mobility Aid 11a
• PROMIS Bank v1.0 - Fatigue (CAT version)
• PROMIS Bank v1.1 - Pain Interference (CAT version)
• PROMIS Bank v1.0 - Depression (CAT version)
• PROMIS Bank v2.0 - Ability to Participate in Social Roles and Activities (CAT version)
• PROMIS Bank v2.0 - Satisfaction with Participation in Social Roles and Activities (CAT version)
