# Supplementary material for: Aging with long-term physical disability: Cohort analysis of survey sample in the U.S
Source: F1000Res. 2023 Dec 14;11:68. Originally published 2022 Jan 20. [Version 2] doi: 10.12688/f1000research.74532.2 (PMC11109573; doi:10.12688/f1000research.74532.2)
Supplement: Peer review history

# Reviewer report: Reviewer response for version 2

**DOI:** 10.5256/f1000research.159782.r259609

**Published:** 2024-05-15

**Authors:** Anthony G Chesebro

**Affiliations:** 1 Department of Biomedical Engineering, Stony Brook University, Stony Brook, USA

As a brief summary of the study: this manuscript provides a succinct cross-sectional analysis of factors affecting AwD, particularly focused on social isolation. One of the greatest strengths of the current study is the effort to recruit participants not typically represented in aging studies. Prior studies of aging and dementia in racially and ethnically diverse cohorts have shown that minoritized racial/ethnic groups experience social stressors that can adversely affect health outcomes. ^1,2^ The current study including outreach focused on enriching their sample for racial and ethnic diversity is to greatly be commended and gives me greater confidence in the generalizability of their results (albeit with the limitations already noted in the discussion).

 In the follow-up longitudinal study that includes T1 and T2, I would be interested in examining:

 1. How stable AwD-related symptoms are during self-report over time. Given that the means are elevated compared to the general population average but not statistically different, I would be curious if the standard deviation shrinks over time (as folks who are aging become more certain of their self-report) or if there is a reversion to the mean. This would not impact the interpretation of the current results as it is already circumspect, but it would give additional insight into these distributions.

 2. The intersection of race/ethnicity report of social engagement and how this compares with the distribution of physical function and health across racial/ethnic groups. Recent work from the same dataset ^3^ has also noted that Black participants have higher participation in social activities compared to White participants, but does not examine the relationship with physical function and health at this intersection. Given the emergence of new trials focused on reducing social isolation as adjuvant healthcare for AwD, ^4^ I think these types of relationships are worth examining to guide future work.

 Overall, I think the study is sufficiently strong and the authors have been responsive to the initial round of review in a way that satisfies any concerns I have. Any further suggestions are for future studies, not this manuscript.

Is the work clearly and accurately presented and does it cite the current literature?

Yes

If applicable, is the statistical analysis and its interpretation appropriate?

Yes

Are all the source data underlying the results available to ensure full reproducibility?

No

Is the study design appropriate and is the work technically sound?

Yes

Are the conclusions drawn adequately supported by the results?

Yes

Are sufficient details of methods and analysis provided to allow replication by others?

Yes

Reviewer Expertise:

Computational neuroscience, Neuroimaging, Aging and dementia

I confirm that I have read this submission and believe that I have an appropriate level of expertise to confirm that it is of an acceptable scientific standard.

## References

[1] : Socioeconomic and psychosocial mechanisms underlying racial/ethnic disparities in cognition among older adults. *Neuropsychology* .2021;35(3) : 10.1037/neu0000720 265-275 10.1037/neu0000720 33970660 PMC8363204

[2] : Brain Aging Among Racially and Ethnically Diverse Middle-Aged and Older Adults. *JAMA Neurol* .2023;80(1) : 10.1001/jamaneurol.2022.3919 73-81 10.1001/jamaneurol.2022.3919 36374494 PMC9664371

[3] : Aging with Disability Symptoms and Ability to Participate in, and Satisfaction with, Social Participation among Persons Aged 45-64. *Healthcare (Basel)* .2022;10(5) : 10.3390/healthcare10050903 10.3390/healthcare10050903 35628040 PMC9141701

[4] : Randomised controlled trial of the Caring Connections intervention to reduce loneliness and perceived social isolation in persons with spinal cord injuries and disorders: study protocol. *BMJ Open* .2022;12(11) : 10.1136/bmjopen-2022-063246 e063246 10.1136/bmjopen-2022-063246 36400737 PMC9677023

# Reviewer report: Reviewer response for version 1

**DOI:** 10.5256/f1000research.78298.r173572

**Published:** 2023-11-21

**Authors:** Ellen Melbye Langballe

**Affiliations:** 1 Vestfold Hospital Trust, Norwegian National Centre for Ageing and Health, Tønsberg, Norway; 2 Department of Geriatric Medicine, Oslo University Hospital, Oslo, Norway

This is a timely and important study. The included original data and statistical analysis are well-described and sufficiently discussed.

 However, a significant weakness of the paper in its present form is that sample integrity, investigated by comparing the results of the original analysis with results reported in existing literature, is not described or discussed in the abstract’s method section, in the methods section of the paper (e.g. how comparable papers were retrieved (systematically search?), number of comparable studies), or in the discussion section (e.g. limitations). Necessary details of the methods used regarding the comparison of results from existing literature should be provided.

Is the work clearly and accurately presented and does it cite the current literature?

Partly

If applicable, is the statistical analysis and its interpretation appropriate?

Yes

Are all the source data underlying the results available to ensure full reproducibility?

No

Is the study design appropriate and is the work technically sound?

Partly

Are the conclusions drawn adequately supported by the results?

Partly

Are sufficient details of methods and analysis provided to allow replication by others?

No

Reviewer Expertise:

ageing, epidemiology, disability, psychology

I confirm that I have read this submission and believe that I have an appropriate level of expertise to confirm that it is of an acceptable scientific standard, however I have significant reservations, as outlined above.

# Response

**Published:** 2023-12-05

**Authors:** Susan Stark

**Affiliations:** Washington University in St. Louis, USA

**Reviewer 2 comment: **A significant weakness of the paper in its present form is that sample integrity, investigated by comparing the results of the original analysis with results reported in existing literature, is not described or discussed in the abstract’s method section, in the methods section of the paper (e.g. how comparable papers were retrieved (systematically search?), number of comparable studies), or in the discussion section (e.g. limitations). Necessary details of the methods used regarding the comparison of results from existing literature should be provided.

**Response:** Thank you for the opportunity to answer these questions about sample integrity. We now see that our description of our process of considering our sample was too brief and also not specific enough. Given the word limitations, we have attempted to provide a brief, but fuller, description of this with the following edits to the manuscript:

 1. We edited the Abstract Methods to add the following sentence: 

*“Results were manually compared against AwD study samples identified through a focused literature review and national census data.”*

 2. At the end of the Methods section, we added a paragraph titled *“Focused literature review and sample comparisons.”* This section describes how we reviewed the literature, which articulates the databases we used and the search criteria. We did not complete a search using a systematic review protocol, but rather identified articles through a focused review that involved pursuing citations of discovered articles in an attempt to find further studies. Because this area of literature is quite small, and often differently indexed with inconsistent keywords, for example, it is difficult to find literature. As we mention in this new section, we found only four separately published studies, but all came from the same parent data source. For that reason, we also drew on U.S. Census Bureau data to consider our sample.

*“ **Focused literature review and sample comparisons***

*Using PubMed and Google Scholar databases, we conducted a focused literature review of peer-reviewed journal articles in English, seeking U.S.-based cross-disability samples (composed of more than a singular disease or impairment diagnosis group) from 2011–2021 of over 100 individuals who had self-reported long-term physical disability, were between the ages of 18 and 65, and who were asked about pain, fatigue, and/or depression using measures similar to the PROMIS measures we used in our study. We located four articles that met these criteria; however, they all used the same or similar data collected at a single university. We reviewed the demographic traits of our sample against the samples used for those four studies and also 2019 U.S. Census Bureau data to better understand differences in our sample compared to the general U.S. and the state-specific populations in the same age range.”*

 3. In the first paragraph of the Discussion section, we changed the wording of the first sentence from *“Findings from our analyses show that our cohort is distinct yet similar to other AwD study populations”* to:

*“Findings from our analyses show that our cohort is distinct yet similar to the other AwD cohort study samples we identified.”*

 We also updated one reference in the first paragraph of this section, replacing Harrison et al. with Walker et al.:

*“…for example, work by Walker et al. ^47^ focuses on predominantly Latina participants”*

 In the fourth paragraph of the Discussion, we modified the first sentence, changing it from, *“Our participants experience common AwD symptoms found in other studies,” *to:

*“Our participants experience common AwD symptoms found in the four studies we identified.”*

 Later in the same paragraph, we added the phrase *“as we understand it at this time”* to the end of the fifth sentence.

 4. In the Study Limitations section, we added two sentences to further clarify the limitations of our analysis:

*“The cross-disability AwD literature is quite small. Our sample comparison was narrow and targeted; it may not fully reflect AwD samples found more broadly in the literature or capture information present in study samples of single-disability, -impairment, or -disease groups, for example. As noted in our introduction, however, pain, fatigue, and depression are commonly found among participants in these studies.”*

 5. In the Conclusions section, to increase clarification and reduce word count (as our edits added words) we rephrased the conclusion of our findings to be more limited in scope, eliminating phrases we believed were not necessary or perhaps redundant, stating:

*“These findings add to the growing body of knowledge about what common traits AwD study populations may have.”*

# Reviewer report: Reviewer response for version 1

**DOI:** 10.5256/f1000research.78298.r173578

**Published:** 2023-07-20

**Authors:** Kenzie Latham-Mintus

**Affiliations:** 1 Indiana University Bloomington, Bloomington, Indiana, USA

In general, there are a limited number of publicly available datasets that enable researchers to examine aging with disability experiences, particularly among those in midlife or late midlife. Likewise, longitudinal data sources are even rarer and typically were not constructed to assess aging with disability experiences. Given these data limitations, this research contributes to the existing literature by creating a *prospective* longitudinal cohort study of midlife (aged 45–65) individuals who are aging with disability. This article outlines the recruitment and data collection processes and analyzes baseline characteristics. The article is well written, and the analysis is appropriate. The research team clearly describes the data limitations of their convenience sample including the over-representation of one state in the US. Most findings are in line with previous research; however, some novel findings (e.g., social participation differences by race/ethnicity) highlight the contributions of this study and the need for more research.

Is the work clearly and accurately presented and does it cite the current literature?

Yes

If applicable, is the statistical analysis and its interpretation appropriate?

Yes

Are all the source data underlying the results available to ensure full reproducibility?

Yes

Is the study design appropriate and is the work technically sound?

Yes

Are the conclusions drawn adequately supported by the results?

Yes

Are sufficient details of methods and analysis provided to allow replication by others?

Yes

Reviewer Expertise:

Sociologies of health, aging, and disability

I confirm that I have read this submission and believe that I have an appropriate level of expertise to confirm that it is of an acceptable scientific standard.

# Response

**Published:** 2023-07-25

**Authors:** Susan Stark

**Affiliations:** Washington University in St. Louis, USA

We thank the reviewer for the time and effort taken to review the manuscript. We appreciate their summary and comments related to our study.

# Response

**Published:** 2023-12-05

**Authors:** Susan Stark

**Affiliations:** Washington University in St. Louis, USA

Thank you for these comments. We greatly appreciate the reviewer's time and feedback.
